# Mother-to-child HIV transmission and its correlates in India: systematic review and meta-analysis

**DOI:** 10.1186/s12884-020-03193-3

**Published:** 2020-09-04

**Authors:** Mihir Bhatta, Nalok Dutta, Srijita Nandi, Shanta Dutta, Malay Kumar Saha

**Affiliations:** 1 Division of Virology, ICMR-National Institute of Cholera and Enteric Diseases, Kolkata, West Bengal India; 2 Division of Bacteriology, ICMR-National Institute of Cholera and Enteric Diseases, Kolkata, West Bengal India

**Keywords:** HIV, HIV prevalence, MTCT, HIV transmission, Women with HIV, HIV-infected infant, PMTCT, India

## Abstract

**Background:**

In India, preventing mother-to-child transmission (PMTCT) of Human Immunodeficiency Virus (HIV) remains one of the foremost challenge in community health. Countrywide MTCT of HIV is estimated to be > 10,000 annually. Aims of present study are to find out the prevalence of HIV and correlates of HIV transmission among children given birth by HIV infected mother through systematic review along with meta-analysis.

**Methods:**

All avaiable articles are retrieved using MEDLINE, Cochrane Library, Science Direct, EMBASE, Google Scholar and PUBMED following guidelines for Preferred Reporting Items for Systematic Reviews and Meta-Analyses (PRISMA). Joanna Briggs Institute Meta-Analysis of Statistics Assessment and Review Instrument (JBI-MAStARI) are applied to critically reviewing the selected articles. STATA 13.0 is used to preparation of forest plot for Meta-analysis. For assessment of heterogeneity and publication biases I^2^ statistics along with Begg and Mazumdar’s test and Egger’s tests are used. Odds ratio (OR) along with forest plots have been showing with 95% confidence interval (CI).

**Results:**

All together 10 studies including 1537 pairs of mothers and new births are assessed in present meta-analysis. Present analysis revealed the prevalence of HIV due to MTCT in India as 8.76% (95% CI; 5.76, 12.31). Analysis of subgroups exhibit a higher pooled prevalence in eastern region of India, 10.83% (95% CI: 5.9, 17.81) and lower in in Western region in India, 6.37% (95% CI: 4.65, 8.49). Status of MTCT before and after initiation of universal ART are 10.23% (95% CI 6.61, 14.55) and 7.93% (95% CI 4.18, 12.76) respectively. Associated factors with MTCT of HIV include absence of maternal prevention of MTCT intervention, OR = 10.82 (95% CI: 5.28, 22.17), lacking in administration of infant ARV (antiretroviral), OR = 8.21 (95% CI: 4.82, 14.0) and absence of medical facility during childbirth OR = 3.73 (95% CI: 1.67, 8.33).

**Conclusions:**

In India, pooled HIV prevalence of MTCT as high as 8.78% (95% CI; 5.76, 12.31) among babies born to infected mothers warrants urgent need of focused intervention for providing ART (PMTCT intervention), ensuring proper infant ARV prophylaxis, and avoiding delivery without proper medical facility to pregnant women with HIV for reduction of occurrence in HIV transmission from mothers to children.

## Background

Since 1980, in developing countries prevalence of HIV has increased and it has led to plenty of economic demographic, and social significances [1, 2]. Primary way to transmission of HIV among adults is chiefly by insecure sexual intercourse. A large incident of vertical transmissions observed from pregnant woman to her new-born during pregnancy in utero, intrapartum during labour and delivery or postpartum through breast feeding [3, 4]. New-born babies are solely infected due to transmission from mother, here considering the parental investment by the male partner, who transmit the infection to his partner, in India this phenomenon is aptly termed as parent-to-child transmission (PTCT) [5]. Seventy to 75 % HIV transmission occurs in the time of labour and delivery, while 25 to 30 % of transmission of infection occurs in the late stage of pregnancy or antenatally [6]. The third largest HIV infected population; approximately 2.39 million are living in India [7, 8]. India has estimated 145,000 children < 15 years of age who are infected by HIV/AIDS [9]. Children account for 7% of all the new HIV infections [10]. National AIDS Control Organization (NACO) estimated that out of about 30 million annual pregnancies in India, more than 22,000 pregnant women are infected by HIV [11]. In the absence of any intervention more than 10,000 infected babies may be born annually [11]. During 2002 PMTCT interventions initiated in India, through free admittance to HIV diagnosis for each enrolled women with pregnancy in nearby antenatal clinics (ANC), administration of a dose of Nevirapine to pregnant women infected with HIV at the time of labour, subsequently also for their children instantly during birth. Free diagnosis of HIV has been made available through integrated counselling and testing centres (ICTCs) associated with ANCs at most government aided healthcare facilities [12]. From 2016 approximately 21 thousand ICTCs are available to offer free services to pregnant women, across India and most are attached with government aided healthcare facilities [13]. Objective of this PPTCT facilities is to prevent the perinatal transmission from a pregnant woman with HIV to her new-born. Programme involves counselling and testing of pregnant women. NACO in India, adopted the WHO recommendations ‘Option B’ [14] for pregnant women, changing from the a dose of Nevirapine to multi-antiretroviral drug prophylaxis strategy. Similarly, on 2013, in accord through WHO strategies [15] and endorsement obtained form national Technical Resource Group [16], NACO initiated to implement the further efficient “Option B+” PMTCT facility which have different constituents intended to improve the health of pregnant women and simultaneously check vertical HIV transmission. The constituents are: providing lifelong ART to each HIV infected woman with pregnancy to avoid vertical HIV transmission and for other added HIV prevention benefits, administration of nevirapine for 6 to 12 weeks to HIV-infected, breastfed children; with engagement and retention of mother and baby in postpartum care of HIV to enable ‘early infant diagnosis’ [17]. Meta-analysis on prevention of MTCT of HIV is scarce and only a solitary article [18] was reported in India context. To gain insight of the Indian scenario the present work of the systematic review and meta-analysis has been initiated on the MTCT of HIV with its correlates in India using accessible published evidence.

## Methods

### Study design and search policy

Present work is the systematic review along with meta-analysis of previously published accessible articles regarding mother to child HIV transmission and its correlates in India. Guidelines for preferred reporting items of systematic reviews and meta-analysis (PRISMA) is followed for present work [19]. Detailed study design is included as Supplementary data.

All published articles are retrieved using appropriate search terms in MEDLINE, Cochrane Library, Science Direct, EMBASE, Google Scholar and PUBMED. We are tried to include all studies which were published during January 2003 to June 2018. The search words are transmission of HIV through mother OR pregnant woman to child/ children OR new-born OR baby/babies OR prevention transmission of HIV from mothers to children OR PMTCT OR elimination of mothers to childlren transmission OR EMTCT OR Prevention of Parent to Child Transmission (PPTCT) AND India.

### Study choice and acceptance criteria

According to the PICOS arrangements (population, interventions, comparisons, outcomes and study design) acceptance criteria have been defined. Present analysis included all accessible published articles directed to evaluate the HIV transmission rate from mothers to children and its correlates in India. All accessible articles are added without confining to a definite study plan. The list containing total number of sorted articles has been checked to retain further studies which could be added in the present analysis. Full text published article in the English are only be accepted.

### Quality evaluation and data accumulation

Evaluation of articles through name, abstract, and entire text of the manuscripts has been performed prior to the addition of it in the ultimate analysis. Critical assessment has been performed with the help of JBI-MAStARI [19, 20]. Standards included in JBI-MAStARI are: study articles are selected in random, proper definition of standard or principle for the addition of article in study, confusing factors are identified and addressed, to estimate the interested outcome, objective criteria are used, consistent calculation of resulting variable and application of reliable statistical methods [19–21]. Critical assessment has been completed before collection of information. Mean score of quality has been used to evaluate quality of encompassed studies. Studies, recorded more than mean of quality score are clustered as higher-quality score and studies are less than the mean score are clustered as the low-quality score.

### Data synthesis

Joanna Briggs Institute (JBI) tool for prevalence studies has been used to perform data synthesis [21]. Every essential information is extracted from the final selected articles by data extraction tool, which bears evidence on author and other details of publication, study area, strategy of study, period of study, time of HIV test for infant, sample size, frequency of MTCT of HIV, and HIV status of infant ARV prophylaxis, availability of medical facility at the time of delivery and status of PMTCT intervention in pregnant women with HIV.

### Noteworthy observations

Principal objective of present article is to find out prevalence of HIV due to MTCT in India. Through PPTCT guidelines, NACO recommends DNA based tests for HIV-exposed infants [3]. DNA based PCR test must be performed at minimum 48 hours of birth to 18 months, otherwise rapid test for detecting HIV antibody after completion of breastfeeding for 6 weeks. When any child is diagnosed with HIV, is generally be referred for further treatment to nearby ART facilities [3, 19]. Moreover, plenty of independent variables are included in present analysis to identify features related with HIV due to MTCT in India. The interdependent features comprised in present analysis is: mother’s ART intervention (ART not received against ART received), infant ARV prophylaxis (not using prophylaxis against use of prophylaxis), place of childbirth (childbirth without proper medical care against childbirth in any minimum healthcare facility). The MoH&FW, Government of India, initiated the program for PPTCT in 2012 [3]. Accordingly, subgroup analysis based on initiation of universal ART in this review has been categorized to exhibit the prevalence of HIV due to MTCT in India.

### Prejudice of publications along with heterogeneity

Heterogeneity of included studies is estimated through I^2^ test statistics along with corresponding *p*-value. The *p*- < 0.05 are used to be termed as heterogeneity. I^2^ statistics of 25% is likely to termed as low, 50% as moderate and similarly 75% is likely to termed as high heterogeneity [19, 22]. Egger’s and Begg and Mazumdar’s assessments are likely to evaluate publication bias, *p* < 0.05 are termed as statistical significance [19, 23, 24]. Nonparametric trim and fill analysis by Duval and Tweedie using random effect has been performed to get results of meta-analysis, which shows non-significant bias in publications (Egger test, *p* > 0.05) [19, 25].

### Statistical approaches towards data analysis

Accumulated information are saved into Microsoft Excel (MS-Office 2019) and then transferred to STATA Ver. 13 (Windows) software to perform meta-analysis [19, 26]. Overall pooled prevalence of HIV due to MTCT in India and its confidence interval at 95% level (CI: 95%) are assed, the prevalence of HIV due to MTCT and the standard error (SE) from each selected study are taken. Forest plots are here applied to represent the pooled prevalence of HIV due to MTCT at 95% CI. Odds Ratio (OR) with CI at 95% level has been represented with forest plot diagram to show the factors correlated with HIV due to MTCT. Subgroup analysis has been performed by study region (western India, eastern India and southern India; here it is noted that no articles on the MTCT of HIV in northern India cannot qualify for the present meta-analysis; study period (before 2010 and after 2010); quality score (low score and high score) of studies and type of the study (prospective cohort and retrospective cohort). The meta-analysis has been performed using a weighted inverse variance random effects model generally used estimating the overall pooled prevalence of the studies are included [19, 22].

## Results

### Study selection

**Fig. 1:**
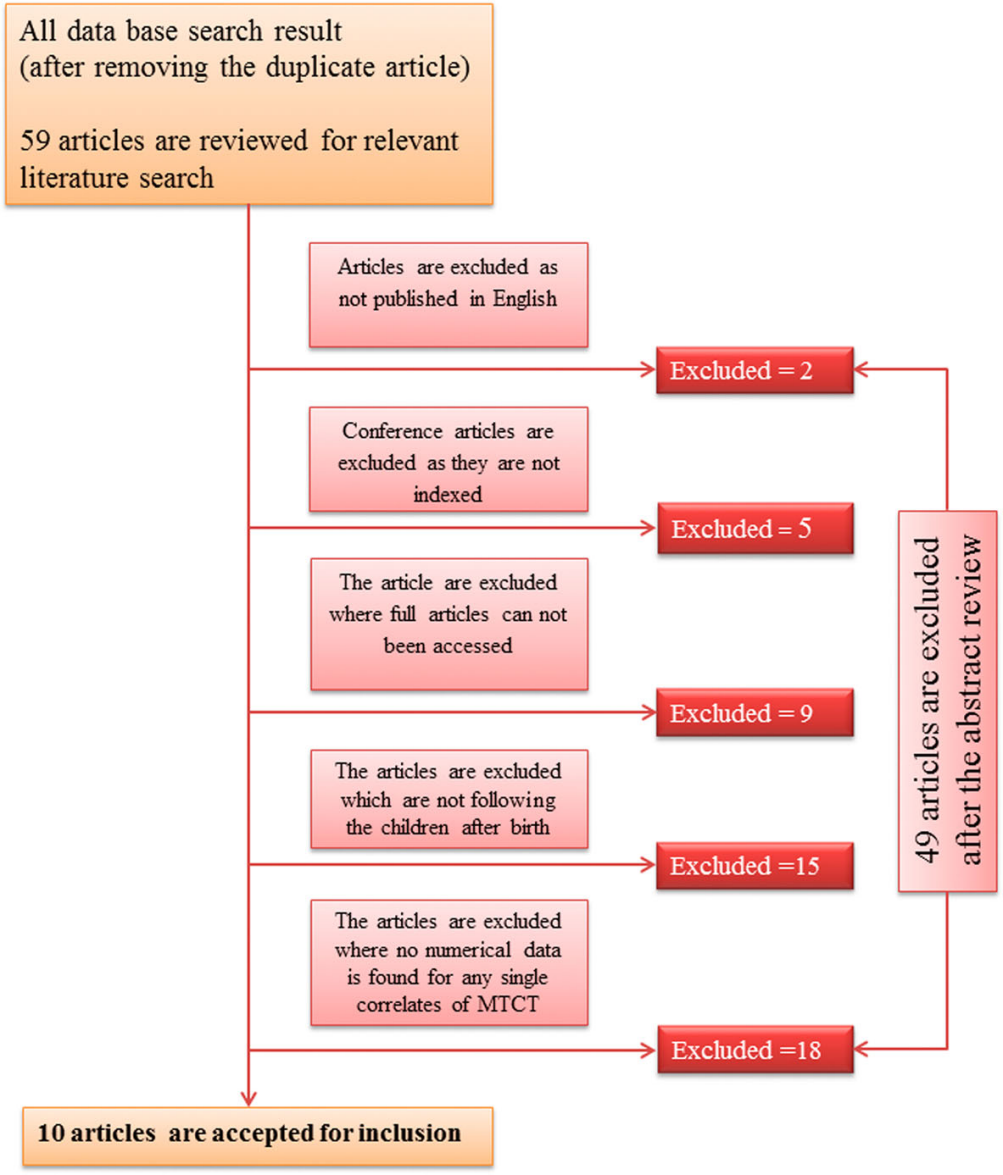
Flow diagram of the studies included in the Meta-analysis

The entire 59 articles are found through web base searching. Articles are sort out through their names, abstracts and entire text. Accordingly, total 49 articles are excluded their name and abstract after reviewing. Ten articles are chosen after reviewing their eligibility. Finally, ten articles are added in present meta-analysis (Fig. 1).

### Publication bias

**Fig. 2:**
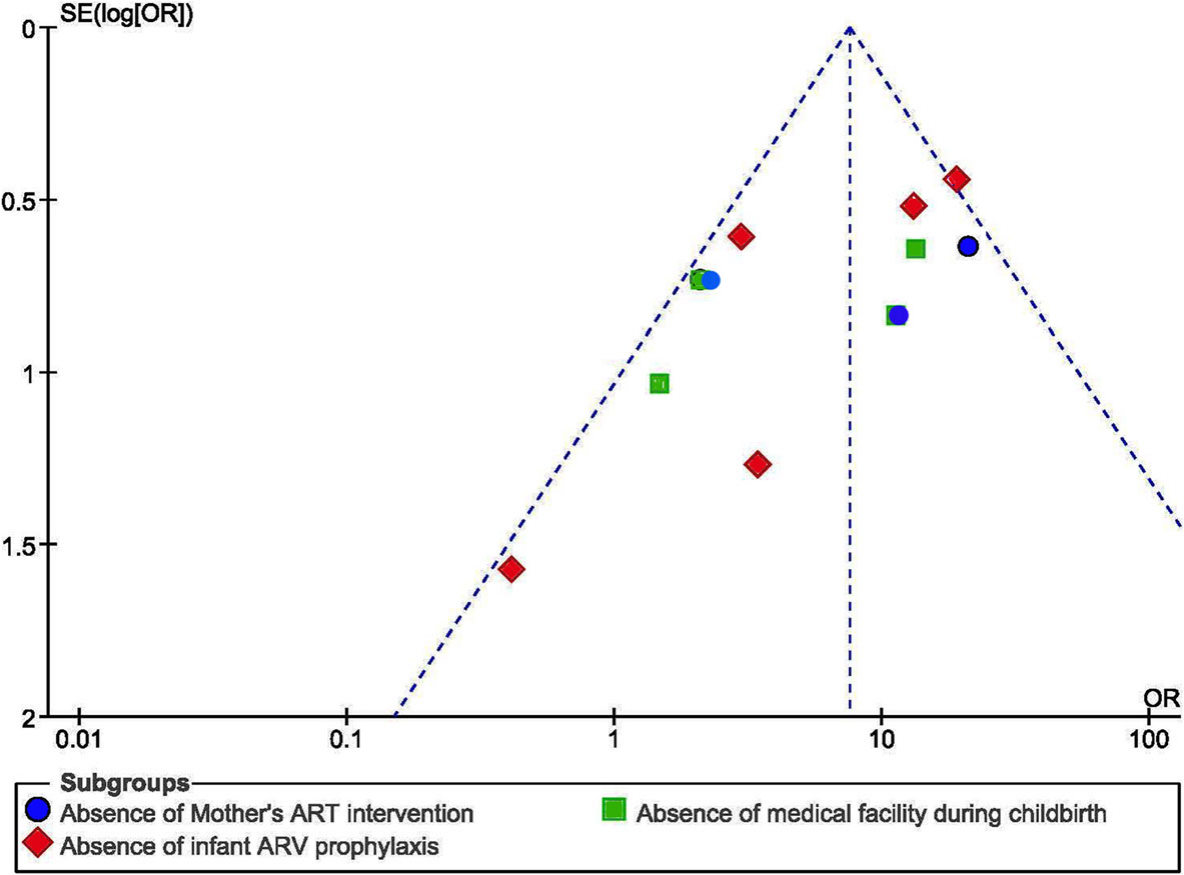
Funnel plot for publication bias, Logprop or LNP (log of proportion) represented in the x-axis

No study was omitted from present prevalence calculation after viewing funnel plot and the significance of Egger’s regression test. Publication bias has been found non-significant with Egger’s regression, *p* > 0.05 observed during present study (Fig. 2).

### Features of included studies

Seven studies, which are included for the final analysis are belong to retrospective cohort [27–33] and three studies are belong prospective cohort [34–36]. The studies used hospital-based PMTCT data and are collected between 2003 to 2018. No published article, has been found after 2013 which could qualify the inclusion criteria for present study. Entire studies including mother-baby pairs, are involved in the PMTCT care in the different health facilities are included. Five studies was taken from Western region of India [27, 29, 30, 33, 34], three studies were taken from Southern region of India [28, 32, 36] and two studies were from Eastern region of India [31, 35]. The sample size ranges from 41 pairs of mothers and children who had a valid HIV test result in Pune, Maharashtra, Western region of India [29] to 362 in Tamilnadu, Southern region of India [36]. Among included studies, total of 1537 pairs of mother and child are included in present mete-analysis (Table 1). Quality score of studies included here having a range from 2 to 10 and mean quality score ± SD is 4 ± 0.71.

**Table 1:** Summary characteristics of studies included in the meta-analysis of the prevalence of mother to child transmission of HIV in India. (where WI: Western region, SI: Southren region and EI: Eastern region in India)

Study	Study area	States	Zone	Study design	Study Period	Sample Size	Time of infant diagnosis	Rate of MTCT of HIV
Ahir et al., 2013 [26]	Department of Microbiology, Seth G.S. Medical College and K.E.M. Hospital, Mumbai	Maharastra	WI	Retrospective cohort study	January 2010 to December 2011	58	48 hours to 24 months	7.143
Bhargav et al., 2012 [27]	Government Hospital Belgaum district	Karnataka	SI	Retrospective cohort study	January 2009 to December 2011	150	2 months to 34 months	8.667
Gupta et al., 2007 [28]	Byramjee JeeJeebhoy Medical College, Pune	Maharastra	WI	Retrospective cohort study	August 16, 2002 to July 8, 2004	41	48 hours to 2 months	9.756
Jhoshi et al., 2010 [29]	Ten (selected) districts in Gujarat	Gujarat	WI	Retrospective cohort study	January 2005 to December 2008	305	up to 18 months	3.571
Chaudhuri et al., 2010 [30]	NRS Medical College and hospital, Kolkata	West Bengal	EI	Retrospective cohort study	January 2004 to December 2007	86	72 hours to 18 months	3.488
Parameshwari et al., 2009 [31]	Department of Experimental Medicine, Dr. MGR Medical University	Tamilnadu	SI	Retrospective cohort study	October 2002 to December 2007	56	48 hours to 6 months	14.286
Phadke et al., 2003 [32]	Byramjee JeeJeebhoy Medical College, Pune	Maharastra	WI	Retrospective cohort study	March 2000 to November 2001	149	48 hours to 2 months	8.78
Pai et al., 2008 [33]	Department of Obstetrics and Gynecology, Mahatma Gandhi Institute of Medical Sciences, Sevagram	Maharastra	WI	Prospective cohort study	January 2006 to September 2006	219	48 hours to 4 months	13.33
Mandal et al., 2010 [34]	Department of Obstetrics and Gynecology, North Bengal Medical College	West Bengal	EI	Prospective cohort study	January 2004 to December 2008	111	up to 18 months	29.412
Mukherjee, 2010 [35]	Three districts in Tamilnadu	Tamilnadu	SI	Prospective cohort study	January 2001 to December 2005	362	up to 1 month	5.292

### Quality of included articles

**Fig. 3:**
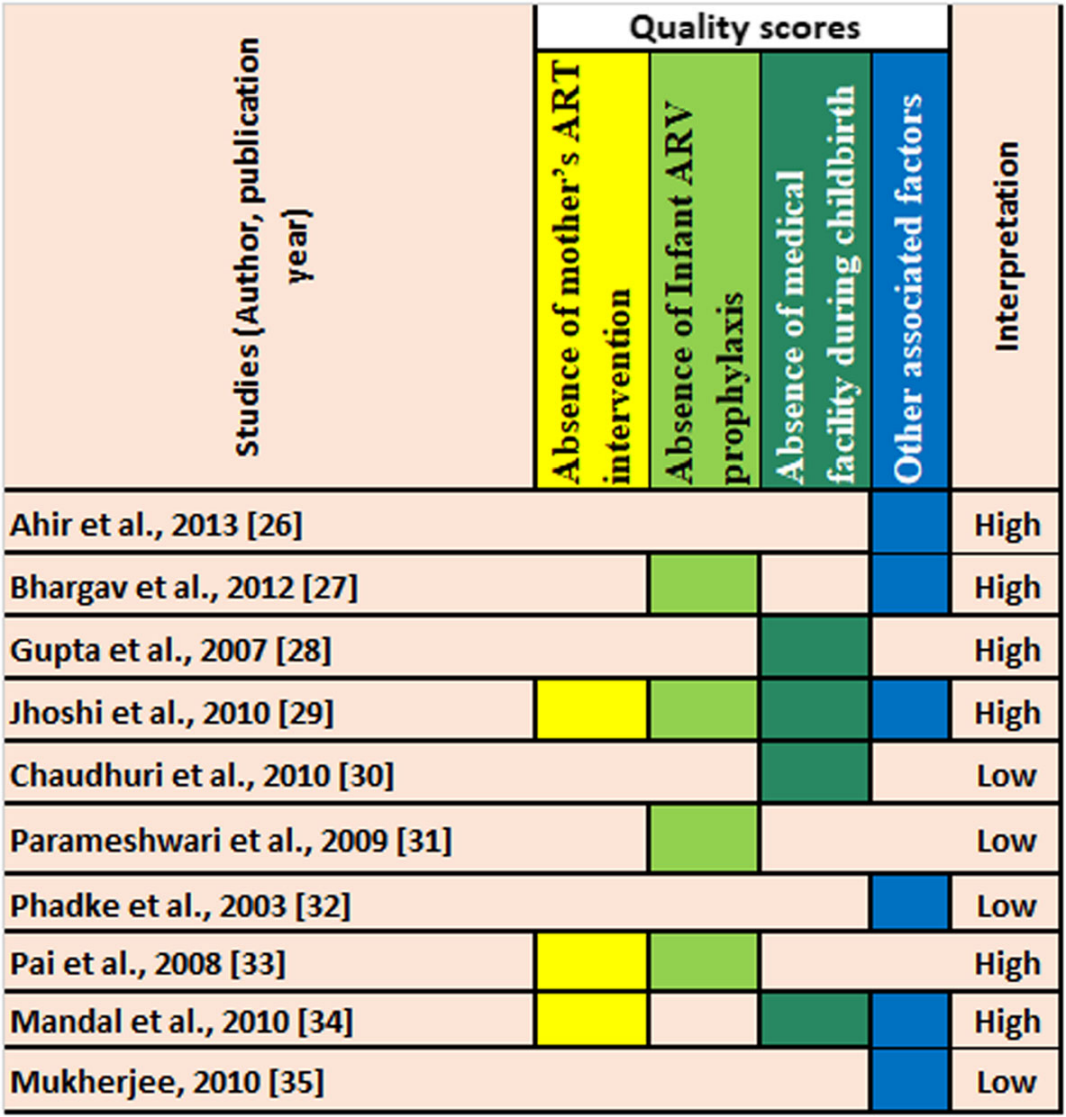
Quality of included studies. (It is noted that, quality of any individual study, which are calculated and presented here is exclusively to fulfill the strains of present study. Authors are not intended to comments on overall quality of any article)

Quality of included studies have been determined and pictorially represented (Fig. 3). It is noted that, quality of any individual study, which are calculated and presented here is exclusively to fulfil the strains of present study. Authors are not intended to comments on overall quality of any article for present study included (Fig. 3).

### Prevalence of HIV due to MTCT in India

**Fig. 4:**
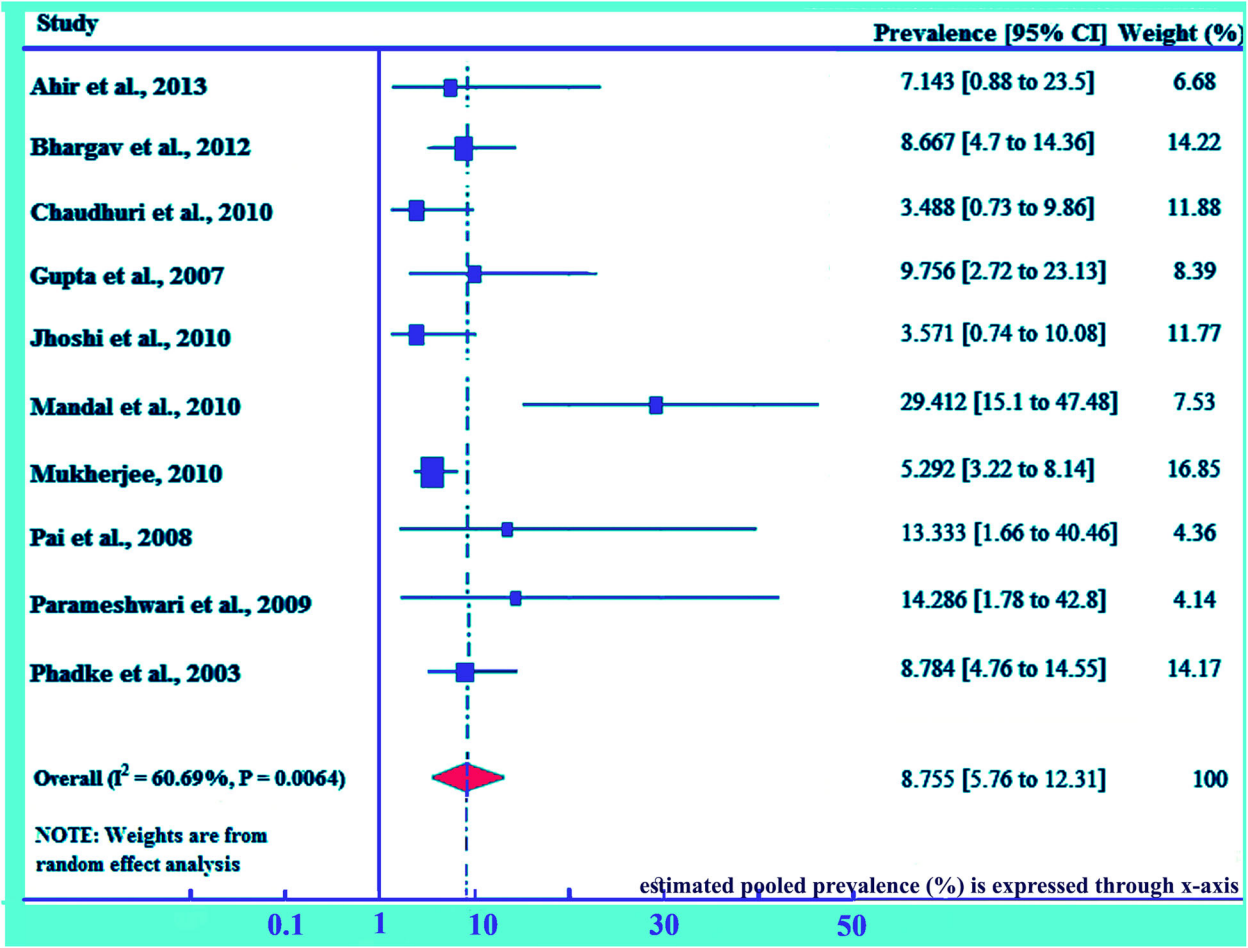
Pooled prevalence of mother to child transmission of HIV in India. The midpoint and the length of each segment indicated prevalence and a 95% CI, whereas the diamond shape showed the combined prevalence

Prevalence of HIV due to MTCT among the studies ranging from as low as of 3.49% (95% CI: 0.725, 9.858) in NRS Medical College and hospital, Kolkata, [31] to as higher as of 29.41% in North Bengal Medical College, both from Eastern region of India [35]. Prevalence of 3.57% in Gujarat, Western region of India [29], 5.29% in District hospital, Tamilnadu, Southern region of India [35], 7.14% in Seth G.S. Medical College with K.E.M. Hospital at Mumbai, Maharashtra, Western region of India [27], and 8.67% in Government Hospital Belgaum district, Karnataka, Southern region of India [28] were also observed. The pooled MTCT of HIV prevalence in India has been estimated to be 8.76% (95% CI; 5.76, 12.31) (Fig. 4). Presence of moderate heterogeneity, I^2^ = 60.69%, *p* = 0.0064 (< 0.01), has been revealed through heterogeneity test. However, publication bias has been detected non-significant with *p* > 0.05.

### Subgroup analysis for MTCT of HIV in India

**Fig. 5:**
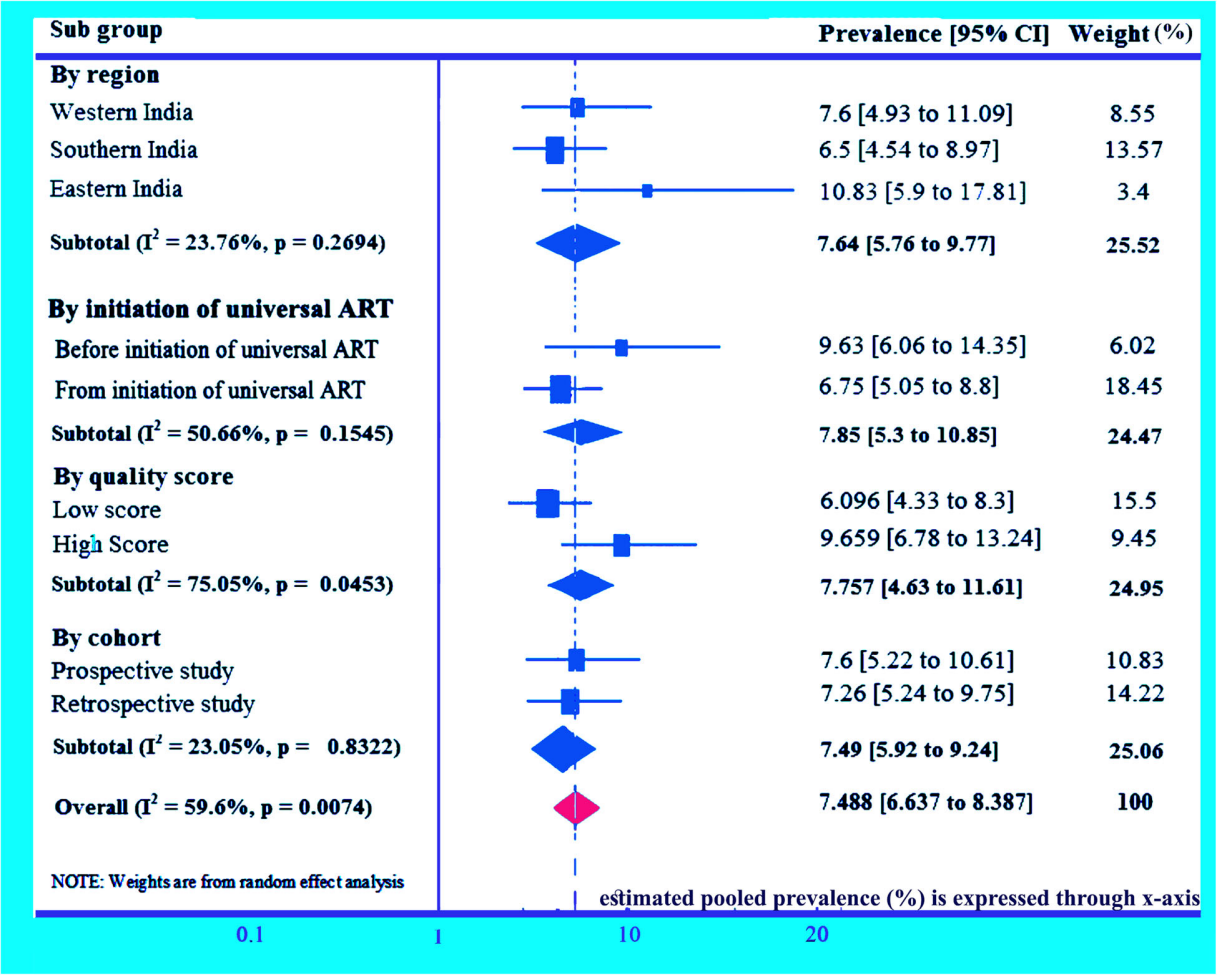
Subgroup analyses (meta-analysis) for the prevalence of mother to child transmission of HIV in India. The midpoint and the length of each segment indicated prevalence and a 95% CI, whereas the diamond shape showed the combined prevalence

Subgroup analysis has been performed through several study characteristics. Subgroup analysis by region shows highest pooled prevalence of HIV due to MTCT in Eastern region in India, 10.83% (95% CI: 5.9, 17.81) and the lowest in Southern region in India, 6.57% (95% CI: 4.54, 8.97). Pooled prevalence of HIV due to MTCT in Western region has been found 7.6% (95% CI: 4.93, 11.09) and I^2^ = 23.73%, *p* = 0.2694. The present result on the pooled prevalence of HIV due to MTCT in different study regions in India suggests that prevalence of MTCT in India is *significantly not region specific*. Before 2010, prevalence of HIV due to MTCT has been found 9.63% (95% CI: 6.06, 14.35) and it reduces to 6.75% (95% CI: 5.05, 8.8) after 2010. Prevalence of HIV due to MTCT in India through quality score of studies has been found 9.66% in studies with high-quality score and 6.1% in studies with low quality score. Prevalence studies with in India by study group (cohort) analysis has been found 7.6% in prospective studies and 7.26% in retrospective studies (Fig. 5).

### Subgroup analysis (based on region)

**Fig. 6:**
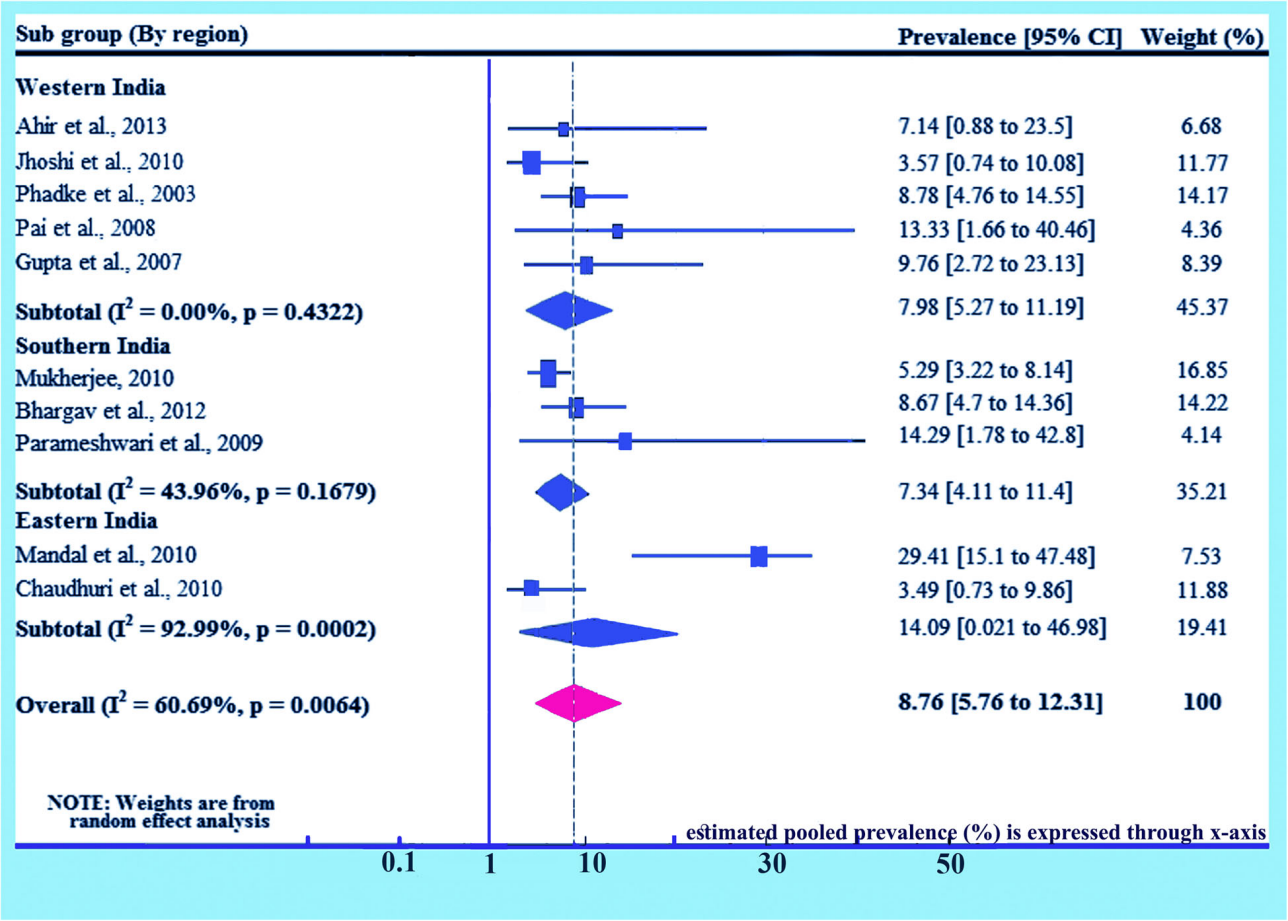
Forest plot of the prevalence with corresponding 95% CIs of the subgroup analysis based on the regions, where the studies done. The midpoint and the length of each segment indicated prevalence and a 95% CI, whereas the diamond shape showed the combined prevalence

Detail analysis on region has been done separately given 100% weightage to prevalence of HIV due to MTCT on the basis of region. Here prevalence for Western India is found 7.98% (95% CI 5.27, 11.19) and I^2^ = 0.0%, *p* = 0.4322, for Southern India is found 7.34% (95% CI 4.11, 11.4) and I^2^ = 43.96%, *p* = 0.1679 and for Eastern India is also found 14.09% (95% CI 0.021, 46.98) and I^2^ = 92.99%, *p* = 0.0002. The overall prevalence has been found 8.76% (95% CI: 5.76, 12.31) and I^2^ = 60.69%, *p* = 0.0064 (Fig. 6).

### Subgroup analysis (based on initiation of universal ART)

**Fig. 7:**
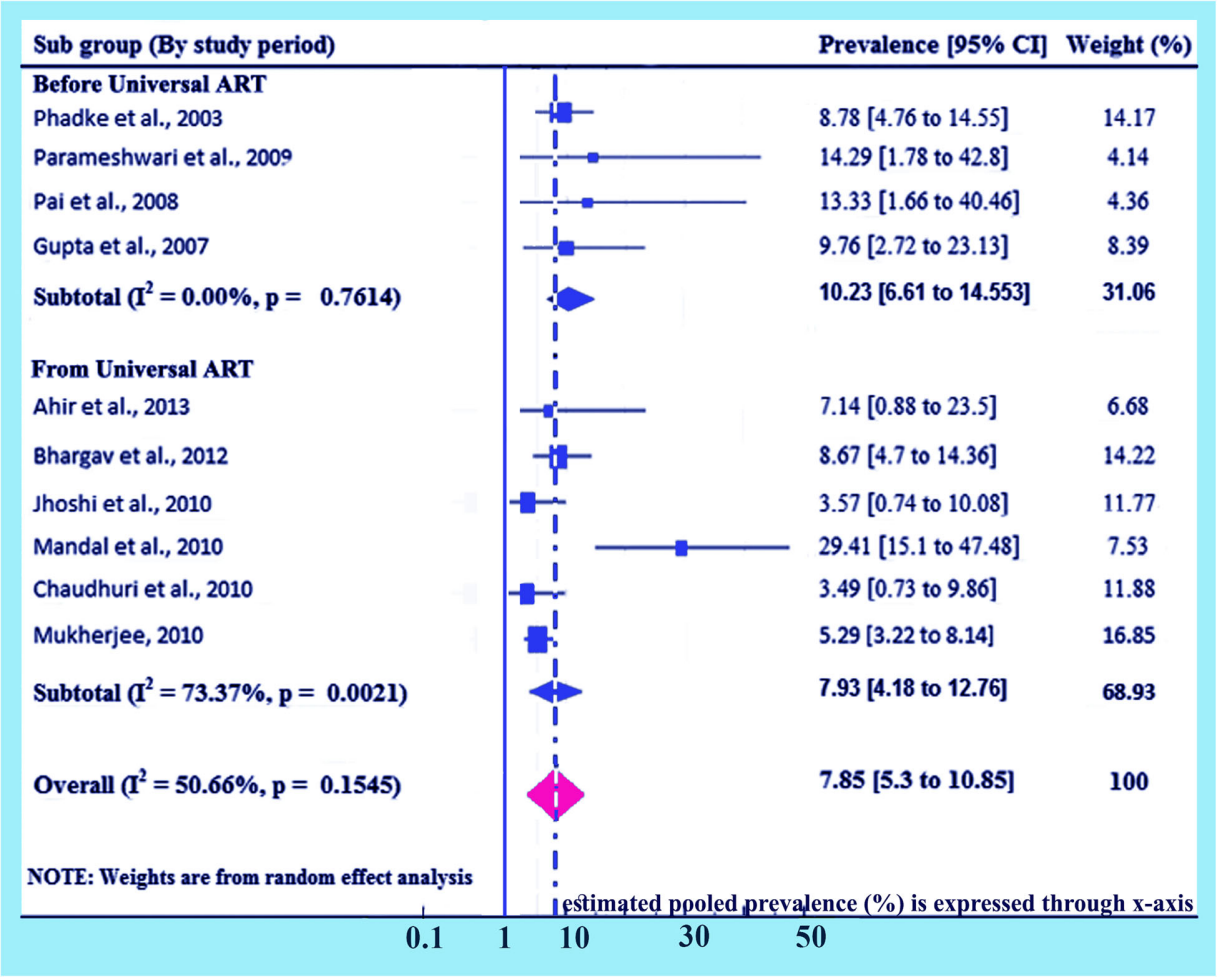
Forest plot of the prevalence with corresponding 95% CIs based on initiation of universal ART. The midpoint and the length of each segment indicated prevalence and a 95% CI, whereas the diamond shape showed the combined prevalence

Detailed analysis on the study period has been done separately given 100% weightage to prevalence of HIV due to MTCT on the initiation of universal ART. Here prevalence for the studies performed before universal ART in India are found 10.23% (95% CI 6.61, 14.55) and I^2^ = 0.0%, *p* = 0.7614, and for the studies performed after initiation of universal ART in India are found 7.93% (95% CI 4.18, 12.76) and I^2^ = 73.37%, *p* = 0.0021. Overall prevalence has been found 7.85% (95% CI: 5.3, 10.85) and I^2^ = 50.66%, *p* = 0.1545 (Fig. 7).

### Subgroup analysis (based on quality score)

**Fig. 8:**
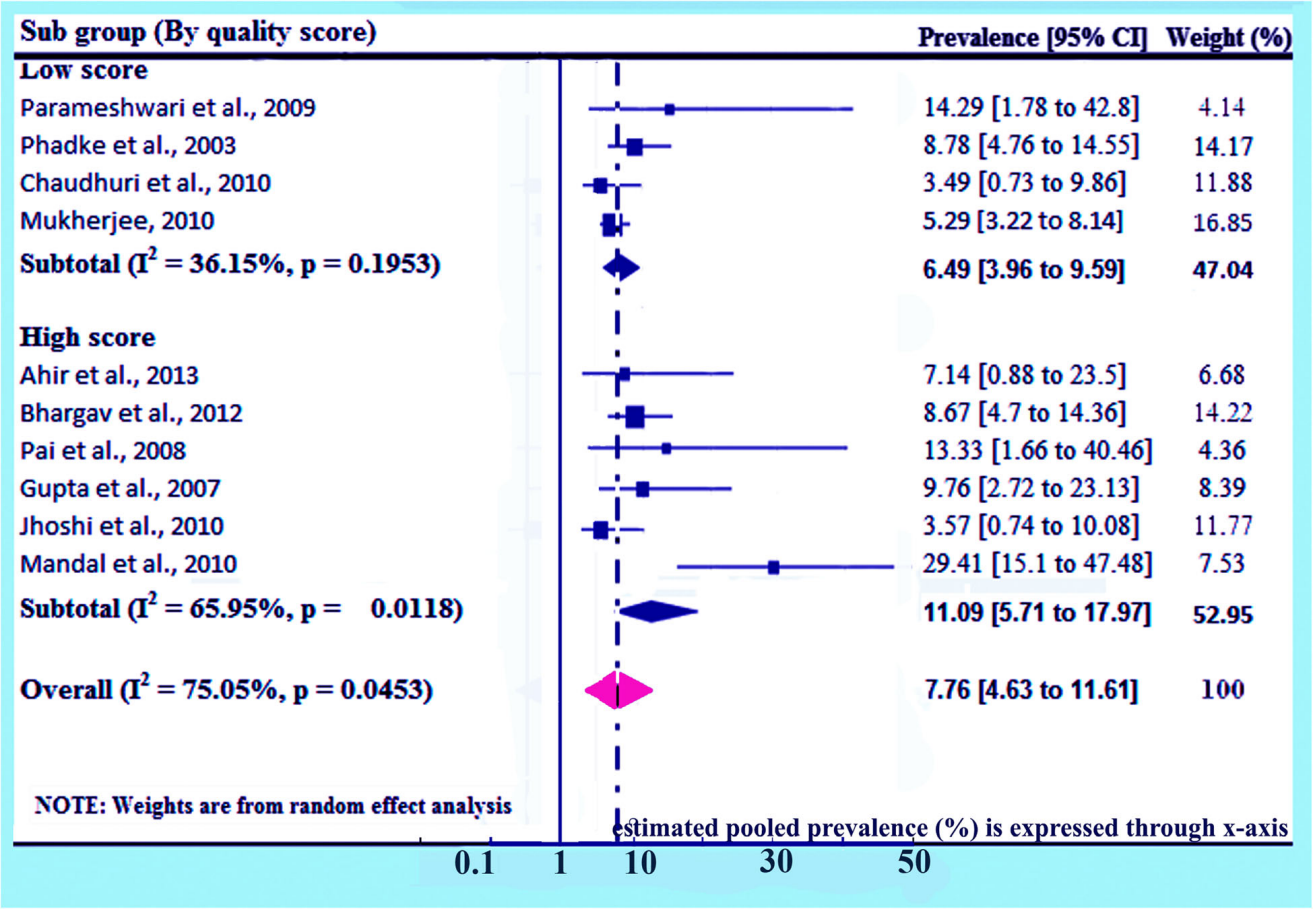
Forest plot of the prevalence with corresponding 95% CIs of the study’s quality score. The midpoint and the length of each segment indicated prevalence and a 95% CI, whereas the diamond shape showed the combined prevalence

Detailed analysis on quality score has been done separately given 100% weightage to prevalence of HIV due to MTCT on basis of quality score. Here prevalence for studies of low scores are found 6.49% (95% CI 3.96, 9.59) and I^2^ = 36.15%, *p* = 0.1953, and for studies of high scores are found 11.09% (95% CI 5.71, 17.97) and I^2^ = 65.95%, *p* = 0.0118. The overall prevalence has been found 7.76% (95% CI: 4.63, 11.61) and I^2^ = 75.05%, *p* = 0.0453 (Fig. 8).

### Subgroup analysis (based on cohort study)

**Fig. 9:**
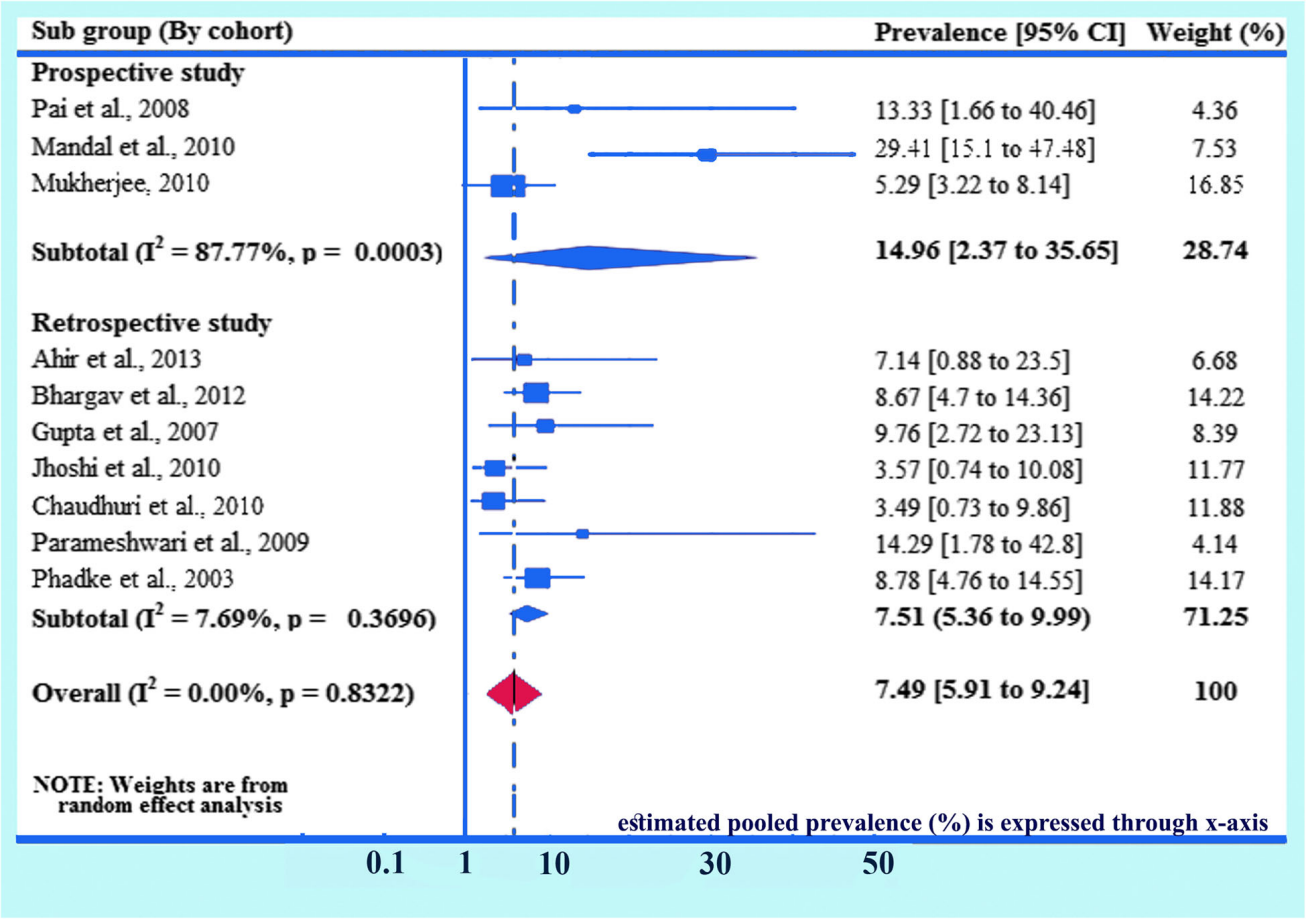
Forest plot of the prevalence with corresponding 95% CIs of the study design (cohort study). The midpoint and the length of each segment indicated prevalence and a 95% CI, whereas the diamond shape showed the combined prevalence

Detailed analysis on cohort study has been done separately given 100% weightage to prevalence of HIV due to MTCT on the basis of cohort study. Here prevalence for prospective studies are observed 14.96% (95% CI 2.37, 35.65) and I^2^ = 87.77%, *p* = 0.0003. Here, only three studies are included as prospective studies, and for the retrospective studies are found 7.51% (95% CI 5.36, 9.99) and I^2^ = 7.69%, *p* = 0.3696. Overall prevalence has been found 7.49% (95% CI: 5.91, 9.24) and I^2^ = 0.0%, *p* = 0.8322 (Fig. 9).

### Absence of ART intervention for mother’s on of HIV due to MTCT

**Fig. 10:**
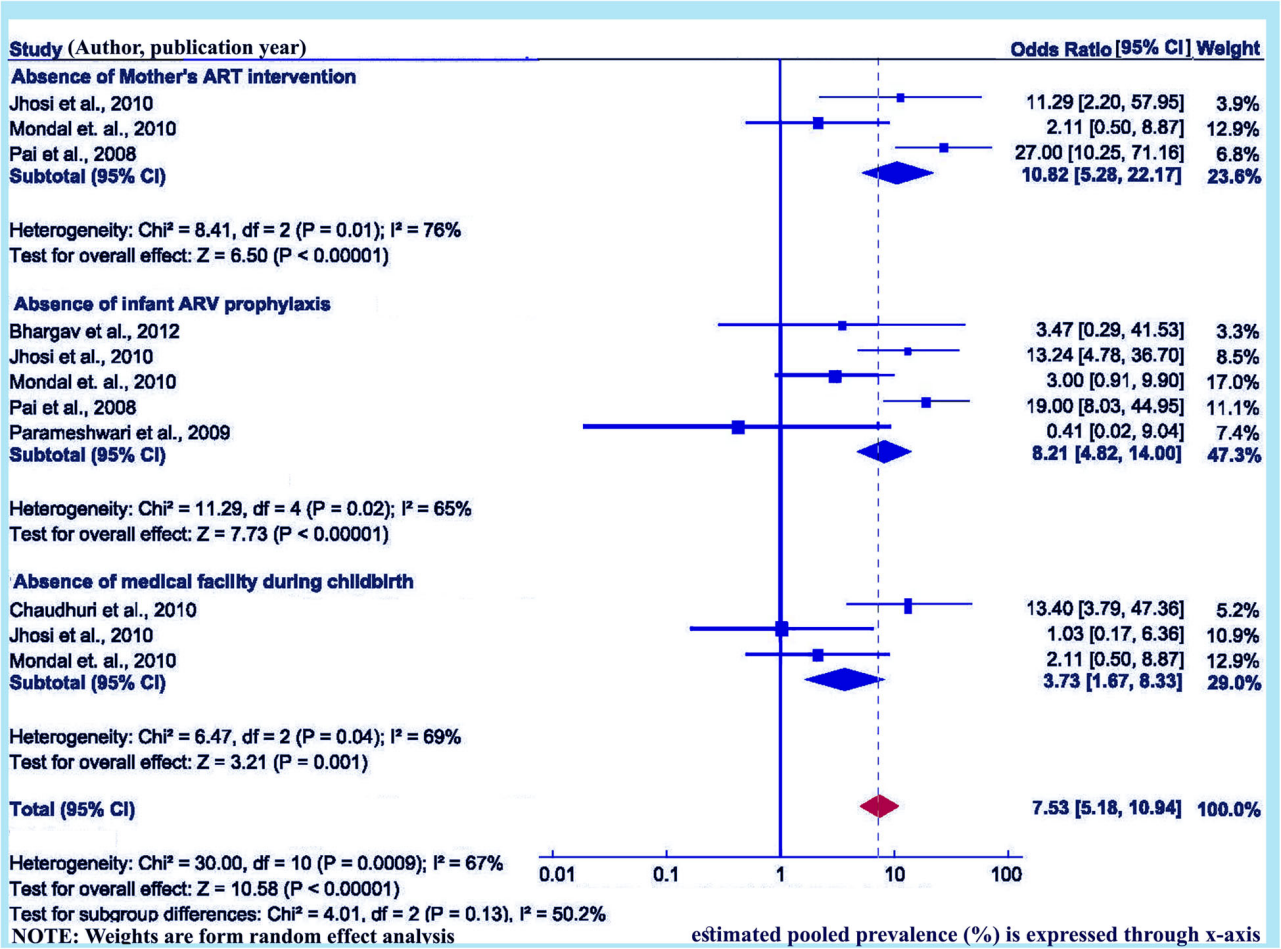
Forest plot displaying the mother to child transmission of HIV and its correlates in India. The midpoint and the length of each segment indicated prevalence and a 95% CI, whereas the diamond shape showed the combined prevalence

Three studies, including 635 pairs of mothers and children, are assessed to determine the relationship of ART intervention with women at the time of pregnancy, or labour and delivery or the postnatal period and risk of HIV transmission to new-born babies [30, 34, 35]. Present meta-analysis revealed that pregnant women who didn’t use the recommended ART intervention/s at the time of pregnancy/ labour and delivery/breastfeeding period are more likely to transmit HIV to their children, OR = 10.82 (95% CI: 5.28, 22.17). Heterogeneity test showed clear evidence of statistically significant high heterogeneity, I^2^ = 76.0% and *p* = 0.01 (Fig. 5). However, publication bias has been non-significant, according to Begg and Mazumdar’s test Z = 6.50, *p* = 0.006 and Egger’s test *p* < 0.00001 (Fig. 10).

### Lack of infant ARV prophylaxis on MTCT of HIV in India

Total five studies including 841 pairs of mothers and children, have been evaluated to assess the relationship of lack of infant ARV prophylaxis with HIV due to MTCT in India [28, 30, 32, 34, 35]. Two studies clearly showed non-significant relation between application of infant ARV prophylaxis with transmission of HIV to new born babies [28, 32]. The rest of selected articles for this category shows statically significant relation. Accordingly, the meta-analysis shows that infants who didn’t receive ARV are more prone to become HIV-infected than infants, who received ARV on time, OR = 8.21 (95% CI: 4.82, 14.0). However, heterogeneity has been observed statistically significant, I^2^ = 65.0%, *p* = 0.02 (Fig. 5). The Begg and Mazumdar’s test and Egger’s test showed no statistical evidence of publication bias, Z = 7.73, *p* = 0.0006 and *p*-value < 0.00001 correspondingly (Fig. 10).

### Lack of medical facility during childbirth with MTCT of HIV

Three studies including pairs of 502 mothers and children, are evaluated in this category [30, 31, 35]. Two of the included studies [31, 35] showed significant relationship between home delivery and a higher chance of HIV transmission from infected mother to the child. Remaining one study showed no such relation [30]. Pooled meta-analysis showed higher chance of HIV transmission from HIV positive pregnant women who delivered at a place where no medical facility is there than women who gave birth in presence of skilled medical technicians at any rural/ urban healthcare facilities, OR = 3.73 (95% CI: 1.67, 8.33). Heterogeneity has been found statistically significant, I^2^ = 69.0%, *p* = 0.04 (< 0.05) (Fig. 5). Whereas, the Begg and Mazumdar’s test and Egger’s tests showed no statistical evidence of publication bias, Z = 3.21, *p* = 0.44 and *p* = 0.001, correspondingly (Fig. 10). Others factors such as efficacy of breast feeding over mixed feeding, vaginal childbirths are observed as a role player in mother to child transmission of HIV [27, 33, 36] in India but some recent findings on MTCT of HIV shows that they don’t possess any major role in MTCT of HIV [17].

## Discussion

In India, transmission of HIV from mother to new-born, in terms of pooled prevalence is as high as 8.76% (95% CI; 5.76, 12.31). Present findings corroborate with published articles including report of NACO on the prevalence of HIV by MTCT in India [10, 13]. This level of MTCT of HIV is far from what the Indian National AIDS Control Program had planned to accomplish in near future [11]. There is a possibility that these carefully studied cohorts included for analysis are likely to better or worse than all the other hospitals and health centers that have not published their data. It is pretty obvious that the studies presented here are extremely likely to have lower transmission rates than all the unstudied populations.

A recent study in China reported lower prevalence of HIV through MTCT as 3.9% (95% CI; 3.2, 4.6%) [37]. Whereas, another work on South Africa reflects higher prevalence (14%) of parent to child transmission of HIV in children lower than 6 weeks with prevalence of 24% among the babies aged between 3 to 6 months [38]. On the other hand, similar study in Ethiopia showed prevalence of HIV by MTCT as 9.93% (95% CI: 7.29, 12.56), which is much closer to the present findings in India [19]. Major difference in prevalence of HIV through MTCT among advanced and emerging countries might be due to variations in the socio-demographic as well as economic profiles of life, availability of anti-retroviral drugs, coverage for health care system and health-seeking nature of the population [39]. Lower acceptance of PMTCT facilities in those days in India might be the cause of higher prevalence of HIV through MTCT [40]. Poor knowledge of HIV/AIDS or other sexual transmitted diseases (STD) among pregnant women, little knowledge on maternal care, psychological problems and community level issues (social taboo; fear to get identified) might be the common barriers for lower acceptance of PMTCT facilities [41]. Consequently, addressing discrete and public level barriers for lower acceptance of PMTCT facility is vital to decrease the higher frequency of HIV due to MTCT in India [42].

WHO recommended for the PMTCT interventions to afford ARV prophylaxis to the infants’ immediately after birth to 6–12 weeks of continuation [19, 43]. The duration of the administration of ARV to the new-borns depends on the status of ART to the HIV infected mothers [44]. The Nevirapine (NVP) prophylaxis was suggested for early 6 weeks duration for breast feeding infants and for 4 to 6 weeks of same ART prophylaxis for non-breastfeeding infants [45]. Meta-analysis of this review showed that children who didn’t obtain ARV prophylaxis at or after birth are more prone to be HIV-infected than a child who received ARV prophylaxis in time. Several studies also revealed importance of infant ARV prophylaxis to prevent HIV transmission from infected mother to her new-born babies [19, 46, 47]. Antiretroviral prophylaxis administrated to children can serve as both pre and post-exposure prophylaxis against HIV infection and it has a major role to protect new born baby from HIV infection [19, 48, 49]. Present analysis also revealed that pregnant women with HIV infection, who gave birth without proper medical care were more likely to have HIV positive child than pregnant women with HIV infection who were attended by competent medical technicians at any healthcare facilities. This is probably due to absence of proper PMTCT interventions simultaneously during labour and delivery for pregnant women who gave birth at home. It is known that children who were delivered without minimum healthcare facility are prone to HIV-infection from mother due to many harmful outdated practices, such as using common blade to amputating umbilical cord, placental blood adulteration, excision of the uvula, unintended practice of circumcision [10]. In contrast, interventions readily accessible at any health care facility includes the following of any normal contamination prevention procedure, following the progress of labour by partograph, application of ARV prophylaxis to make practices of harmless delivery for the HIV positive women who attended any public health care facility [49]. In India, generally women with HIV are advised for cesarean delivery, which can be carried out only through organized healthcare facility. For an elective cesarean operation, a three to 4 hours window is required to deliver interferences and also permit ART drugs to attain highest tighter in the maternal circulation. This time window may be varying according to the patient’s current health condition and the period of labour. For child birth, however, ART drugs are administered in established labour in the initial stage [50]. Studies performed in Africa and Europe similarly exhibit that a baby born through elective cesarean delivery can strongly prevent HIV transmission from mother [19, 38, 51, 52, 53].

For reduction in the prevalence HIV-positive child, application of PMTCT intervention (providing ART) at the time of pregnancy, during labour, child birth, and breastfeeding period is vital [53, 54]. Findings of this analysis highlighting that pregnant women with HIV infection and devoid of antiretroviral treatment are more prone to give birth of a child with HIV. It is estimated that without administration of antiretroviral treatment, 20 to 45% of new-borns may be affected with HIV globally [11, 13, 14, 19]. In contrast, antiretroviral drugs could reduce the viral load in mother, and hence lower the chance of HIV transmission [12, 13]. According to the WHO, ART interventions to the pregnant woman including her new-born could lower the chance of mother to child transmission of HIV less than 2% [16]. However, very young maternal age [31], HIV infected women, who didn’t avail antenatal care service [33], late admission or without admission of HIV infected infant for follow up in public health care system [34], small period of ART [26], lower maternal CD4 count [32], and low child birth weight (less than 2.0 kg in India) [32], inappropriate family planning (FP) [18, 19] may be the associate factors for MTCT of HIV in India, which warrants further research and surveillance. Also, the resulting attributes might be affected by other lurking variables, which are not stated in the present work [54, 55].

### Limitations of the study

Present systematic review and meta-analysis following the PRISMA guideline to includ only qualified articles on MTCT of HIV and excluded unpublished research works and several reports by governmental and non-governmental agencies, articles published in locally available journals which are not indexed in most databases. Several conference articles and abstracts which are not indexed so, are not included in the present study. Absence of any published study from northern India limits pan Indian scenario in this analysis. Present analysis included only a few variables correlates with MTCT of HIV in India, due to limited number of published articles. Only those articles published in English (UK or US) are included in the present study, and these all together may have led to potential reporting bias.

## Conclusions

Indian AIDS control program has made progress in reducing HIV transmission from infected pregnant woman to her new-born babies though country is far away to achieve total elimination of MTCT of HIV. Intervention program strengthening early maternal ART, initiation of ARV prophylaxis of babies born to HIV infected mothers simultaneously institutional caesarian delivery might help in reducing MTCT HIV risk. However, at present more surveillance data are crucial to follow the development of PMTCT program.

## Supplementary information

**Additional file 1: Supplementary file 1.** PRISMA check list.

## Data Availability

All data generated or analyzed are included in this article.

## Abbreviations

ART: Antiretroviral therapy
ARV: Antiretroviral
CI: Confidence interval
DNA: Deoxyribonucleic acid
EMTCT: Elimination of mother-to-child transmission of HIV
HIV: Human immunodeficiency virus
MTCT: Mother-to-child transmission
MTCT HIV: Mother-to-child transmitted HIV
NACO: National AIDS Control Organization
OR: Odds ratio
PCR: Polymerase chain reaction
PMTCT: Prevention of mother-to-child transmission
PTCT: Parent-to-child transmission
PPTCT: Prevention of parent-to-child transmission
WHO: World Health Organization
